# Effect of orthodontic treatment involving first premolar extractions on mandibular third molar angulation and retromolar space

**DOI:** 10.4317/jced.53434

**Published:** 2017-03-01

**Authors:** Luz-Victoria Mendoza-García, Esther Vaillard-Jiménez, Araceli García-Rocha, Carlos Bellot-Arcís, Vanessa Paredes-Gallardo

**Affiliations:** 1 Master in Orthodontics. Benemérita Universidad Autónoma de Puebla. Puebla, Puebla México; 2 Master in Pediatric Dentistry, Professor, Faculty of Dentistry, Benemérita Universidad Autónoma de Puebla, Mexico; 3 PhD, D. Master in Oral Prosthodontics and Specialist in Oral Rehabilitation. Professor, Faculty of Dentistry, University of Veracruz, Mexico; 4 PhD in Dentistry. Master in Orthodontics UV. Associate Professor, Faculty of Medicine and Dentistry, University of Valencia, Spain; 5 PhD in Dentistry. Master in Orthodontics UCM. Professor, Faculty of Medicine and Dentistry, University of Valencia, Spain

## Abstract

**Background:**

Third molars present more problems than other teeth because they are the last teeth to erupt, and so it is important to assess their development when designing an orthodontic treatment plan. The aim of this study was to compare the angulation of the mandibular third molar and retromolar space before and after orthodontic treatment in cases involving first premolar extraction.

**Material and Methods:**

76 patients, 59 women (77.63%) and 17 men (22.36%), were recruited from the Orthodontics Clinic at Benemérita Universidad Autónoma de Puebla (Mexico). Panoramic radiographs were analyzed before and after orthodontic treatment that included first premolar extractions, measuring retromolar space (RS) and the angles formed by the intersection of the axes of the third and second molar (α) and the intersection of the axis of the mandibular plane and third molar (β).

**Results:**

The data obtained underwent statistical analysis. The angle α and β showed statistically significant differences on the left side in women. In men, only the right side α angle showed significant differences. Retromolar space increased significantly on both sides for both sexes.

**Conclusions:**

Third molar angulation presents different behaviors between men and women, with greater verticalization in women.

** Key words:**Third molar, retromolar space, orthodontics.

## Introduction

Of all the teeth, third molars present the most eruption problems. They are often impacted or retained (1), which can cause conditions such as pericoronitis, cavities, root resorptions, cystic processes, or tumors (2,3).

There is often insufficient space for them to erupt, and so they develop at a more distal site (4) than they would normally occupy in the arch. The roots often show signs of displacement because of distal curvature (5).

Sometimes, third molars are ignored when drawing up an Orthodontic treatment plan; it is assumed that they will not erupt, a situation that will indicate their removal. But it is important to conserve all dental organs and to maintain maximum masticatory function as a key treatment objective. Nevertheless, it has been estimated that 54% of mandibular third molars are removed prophylactically, even though they do not present any subjective symptoms (6).

The World Health Organization (WHO) claims that the presence of at least 20 teeth is desirable; fewer will cause a greater tendency to decay and lower numbers of dental organs induce xerostomy. When teeth are extracted in the course of orthodontic treatment, it cannot be foreseen whether or not the patient will take care of the remaining teeth and conserve all of them in the future.

Third molar root formation starts at the age of 15 and the tooth erupts at the age of 20; if formation is delayed, this will produce a risk of impaction (7). Third molar impaction is associated with a range of pathologies, and so the presence and development of these teeth should be monitored by means of panoramic x-rays (8). In this context, several authors have assessed the accuracy of panoramic radiography for diagnostic purposes, finding it an “accurate method (9-13).

In 1987, M. Richardson observed an increase in retromolar space after a five-year follow-up in a group of patients. He hypothesized that this was due to remodeling in the retromolar area and to mesial movement of the first molar (14).

When the third molar is positioned at an adequate angle relative to the mandibular plane and the longitudinal axis of the second molar, it maintains the necessary force for remodeling and expansion in various dimensions by bone resorption in the region of the mandibular ramus (15). The third molar continually changes its angular position and undergoes a pre-eruptive rotational movement; this rotation occurs as the third molar approaches the second molar. There is an average change of 11.2º in third molars among patients aged 10-15 years old with respect to the mandibular plane unless this movement takes place, impaction will occur (16). Artun (2005) described a favorable change in third molar angulation among patients who underwent extractions for orthodontic treatment purposes, especially in the last stage of root formation (17). Cryer describes a decreased probability of mandibular impacted third molars in patients who underwent premolar extraction prior to orthodontic treatment (18).

The aim of this study was to determine changes to third molar inclination in relation to the mandibular plane and the longitudinal axis of the second molar, comparing angles before and after orthodontic treatment involving first premolar extractions.

## Material and Methods

This observational, analytic, comparative, retrospective, longitudinal study reviewed 2,000 medical records of patients treated at the Department of Orthodontics at Benemérita Universidad Autónoma de Puebla (Mexico), making use of radiographic records of optimal quality.

Radiographs corresponding to 76 subjects were selected for analysis, 59 women (77.6%) and 17 men (22.4%). The mean patient age recorded at the start of orthodontic treatment was 19.67 years. First premolar extractions were indicated in all cases, in response to moderate crowding (4-7mm) to be treated with reciprocal closure. Mandibular third molars were present in all patients before and after orthodontic treatment.

Exclusion criteria were as follows: patients with erupted third molars at the start of treatment, any presence of malformation, syndrome, missing teeth, or supernumerary teeth.

Radiographs of cervical vertebrae and skull lateral x-rays were analyzed to determine each patient’s growth stage (19).

**Figure 1:**
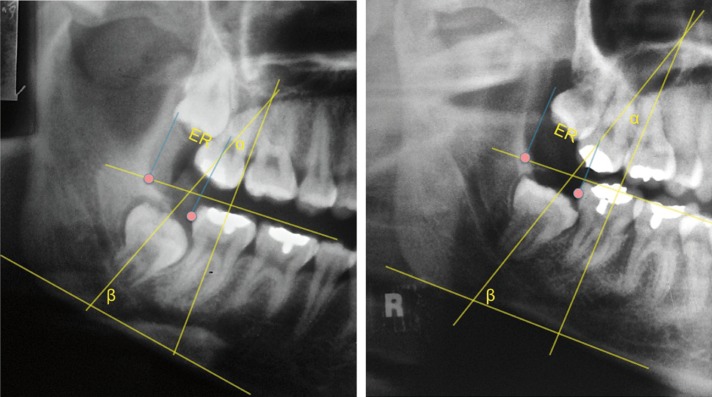
Measurment before and after orthodontic treatment.

The positions of third molars were analyzed in panoramic x-rays taken before and after orthodontic treatment. The following series of linear and angular measurements were made (as described by Turkoz 201315) (Fig. 1).

• Angle α: intersection of the longitudinal axes of the third molar and the second molar.

• Angle β: intersection of the longitudinal axis of the third molar and the mandibular plane.

• Retromolar space (mm) is the distance from point J to point D7. Point J is the point of intersection of the occlusal plane and the anterior surface of the mandibular ramus. Point D7 marks the most distal surface of the second molar.

One month after registering the first set of measurements, 20 x-rays were measured again. Reproducibility measures achieved an intra-class correlation coefficient (ICC) of over 0.90. A second examiner performed the same measurements to check reliability, obtaining an intra-class correlation coefficient (ICC) that ranged between 0.85 and 0.95 for all measurements.

Student’s T test was used to determine the existence of significant differences between means. The level of significance was set at *p* = 0.05. Data were processed using Microsoft Excel and the SPSS statistical software package, version 20.

## Results

 Table 1 shows mean values of all the variables studied. Significant changes were fround in the retromolar area, indicating increases in this area on both sides.  Table 2 shows data comparisons between women and men. In women, the mean α angle showed a decrease on both sides, which was more evident on the left side. The β angle showed an increase on both sides, suggesting third molar verticalization. Retromolar space increased on both sides. In men, there was an increase in the α angle, which was greater on the right side, while the β angle underwent a decrease; together, these data suggested a mesioangulation of the lower third molar on the right side.

**Table 1:**
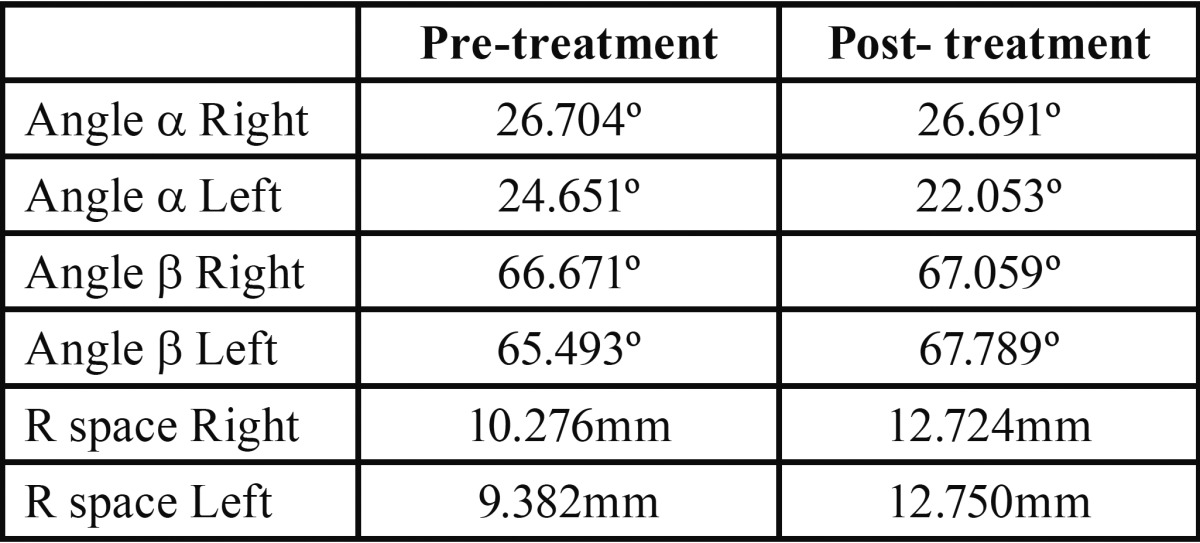
Mean measurements taken before and after orthodontic treatment.

**Table 2:**
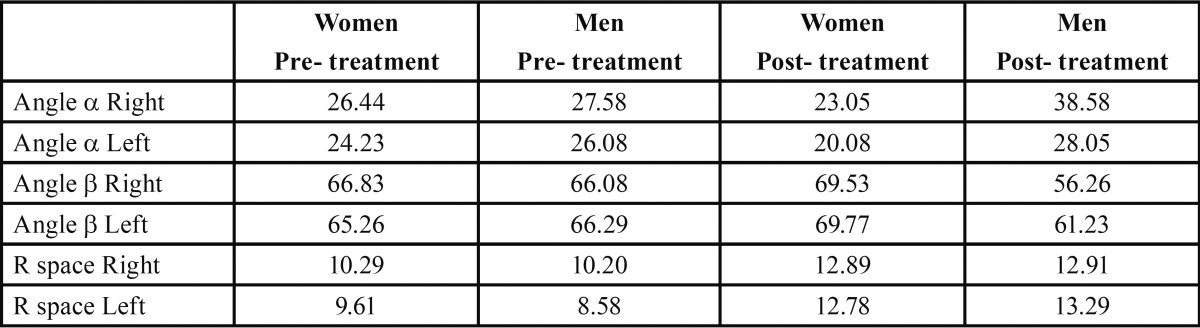
Mean measurements taken before and after orthodontic treatment of women and men.

Student’s t-test identified significant differences in mean values registered before and after treatment: in the α angle (*p*=0.033) and β angle (p=0.016) on the left side in women; in the α angle (*p*=0.030) on the right side in men; retromolar space in both women (*p*=0.000) and men (*p*=0.016 on the right side and *p*=0.000 on the left right), as shown in  Table 3.

**Table 3:**
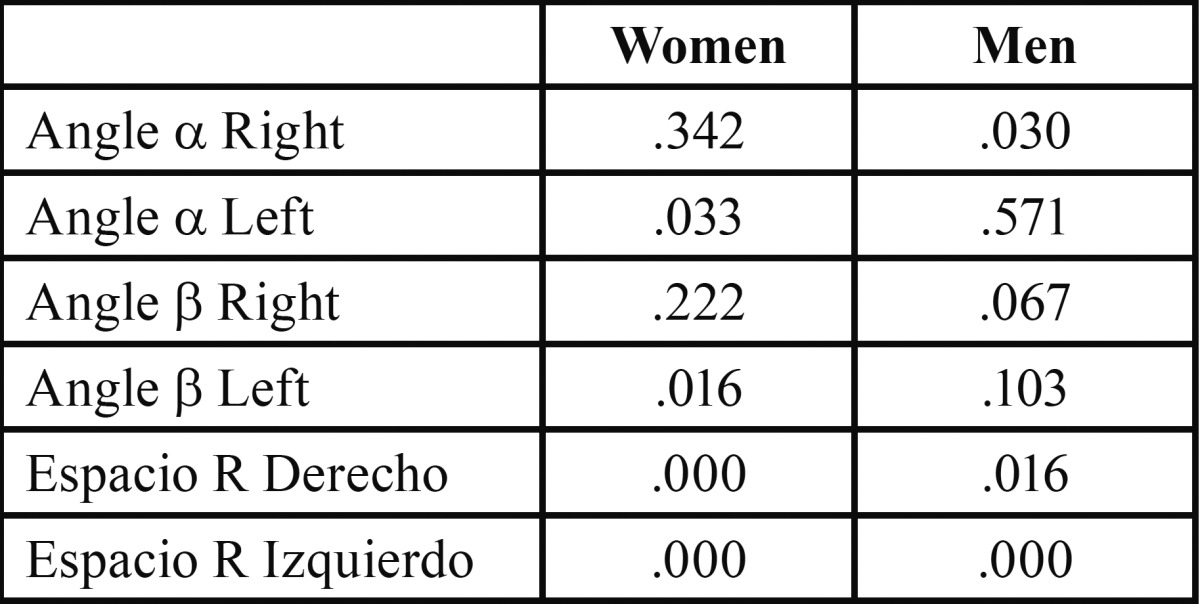
Student’s T test comparing mean values before and after treatment in men and women. odontic treatment of women and men.

## Discussion

For the sake of better orthodontic planning, it is important to determine the future of third molar position on the basis of clinical observation. The findings detailed below place the present results within the context of previous research into this issue, and help provide a fuller picture of the effects of first premolar extraction on mandibular third molar angulation and retromolar space.

-Gender

Analyzing the data obtained by gender, a significant difference was found for men in the α angle on the right side, and in the α and β angles in women on the left side, pointing to verticalization of the lower third molars among women, while these teeth underwent mesioangulation in men.

No other studies to date have divided data by gender, although most previous study samples, like the present study, have included more women then men.

Angle α 

Saysela (20) (2006) and Mihai (21) (2013) looked at changes to the angle formed by the intersection of the longitudinal axes of the second and third mandibular molars. In the present study, significant differences were found in the α angle with the exception of the right side in men, indicating an increase in mesial angulation in men, and on the left side in women, which, unlike men, reflects a decrease which can be assumed to represent verticalization of the lower third molar. The changes observed among female subjects coincide with Saysela and Mihai who observed greater changes in angulation in a group of patients, who underwent extractions of first premolars, suggesting third molar verticalization.

Türköz *et al.* mention that if the α angle is greater than 30º, the third molar shows a greater tendency towards impaction, with the result that fewer impactions were observed in the patient group who underwent extractions (15). In the group of male subjects, mesioangulation of the third mandibular molar was observed, this being significantly greater on the right side. However, some authors such as Jain (22) (2009) and Ahmed (23) (2011) have found large differences in this angle between patients regardless of whether or not they had performed premolar extractions.

Angle β

The β angle also showed some significant changes, an increase among women, and a reduction among men. Kuwari (24) (2013) also noted an increase in this angle in patients who had undergone first premolar extractions. Tarazona (25) (2010) described an improvement in third molar angulation over time, although no significant changes were found between patients who had under-gone premolar extractions and those who had not.

Türköz *et al.* (15) claim that if a patient presents a β angle of less than 60°, he/she will have a greater tendency towards third molar impaction. This author observed fewer impactions among patients who had undergone premolar extractions, as well as verticalization of the third molar. This concurs with the present study, which found that this angle was greater than 60º among women (69.53º on the left, 69.77º on the right), coinciding with greater verticalization than in the group of male subjects, who presented a β angle of less than 60º on the right side.

Ahmed (23) (2011) and Gohilot (26) (2012) did not observe changes to third molar angulation in patients, regardless of whether or not they had undergone extractions.

-Retromolar space 

The present study observed increases in retromolar space on the right side in both men (4.2 mm) and women (2.58 mm). On the left side, retromolar spaces also increased for both sexes, a significant increase of 6.08 mm in men, and 2.1 mm in women.

Richardson (16) (1970)describes an increase in retromolar space of approximately 4mm due to a physiological mesial movement of the teeth; Türköz (15) (2013) also observed increased retromolar space in patients who underwent extractions. Retromolar space has been measured on the basis of varying benchmarks: Kim (27) (2003) measured from the Xi point to the distal face of the third molar on lateral x-rays, describing greater increases in this space among a group of patients who had undergone extractions.

However, other studies claim that third molar angulation is not associated with orthodontic treatment involving extractions (23,28).

The present study suffered several limitations. The study sample was small, as few patients met the inclusion criteria; most of the 2,000 cases reviewed had undergone third molar extraction before finishing orthodontic treatment. Furthermore, the sample was not compared with a control group.

From the results obtained it may be concluded that the changes resulting from orthodontic treatment involving premolar extractions are as follows:

• Verticalization of the third molar in the group of female subjects, represented by the α angle (.033), and the β angle (.016) on the left side.

• In men, unlike women, third molar angulation showed an increase to mesial inclination; changes to the α angle were significant on the right side (.030).

• Retromolar space increased significantly post-treatment for both men and women; the change was greater among men than women.

• Differences were observed in third molar angulation between men and women, whereby the third molar was verticalized in women, while inclination increased among men.
